# Explainability as the key ingredient for AI adoption in Industry 5.0 settings

**DOI:** 10.3389/frai.2023.1264372

**Published:** 2023-12-11

**Authors:** Carlos Agostinho, Zoumpolia Dikopoulou, Eleni Lavasa, Konstantinos Perakis, Stamatis Pitsios, Rui Branco, Sangeetha Reji, Jonas Hetterich, Evmorfia Biliri, Fenareti Lampathaki, Silvia Rodríguez Del Rey, Vasileios Gkolemis

**Affiliations:** ^1^Center of Technology and System (CTS), Instituto de Desenvolvimento de Novas Tecnologias (UNINOVA), Intelligent Systems Associate Laboratory (LASI), Caparica, Portugal; ^2^Knowledgebiz Consulting, Almada, Portugal; ^3^AiDEAS, Tallinn, Estonia; ^4^Institute for the Management of Information Systems (IMSI), ATHENA RC, Athens, Greece; ^5^UBITECH, Athens, Greece; ^6^Fraunhofer-Institut für Offene Kommunikationssysteme (FOKUS), Berlin, Germany; ^7^Suite5 Data Intelligence Solutions, Limassol, Cyprus; ^8^Asociacion De Empresas Technologicas Innovalia, Bilbao, Spain

**Keywords:** explainable AI, XMANAI platform, manufacturing industry, business value, decision-making, Fuzzy Cognitive Maps

## Abstract

Explainable Artificial Intelligence (XAI) has gained significant attention as a means to address the transparency and interpretability challenges posed by black box AI models. In the context of the manufacturing industry, where complex problems and decision-making processes are widespread, the XMANAI platform emerges as a solution to enable transparent and trustworthy collaboration between humans and machines. By leveraging advancements in XAI and catering the prompt collaboration between data scientists and domain experts, the platform enables the construction of interpretable AI models that offer high transparency without compromising performance. This paper introduces the approach to building the XMANAI platform and highlights its potential to resolve the “transparency paradox” of AI. The platform not only addresses technical challenges related to transparency but also caters to the specific needs of the manufacturing industry, including lifecycle management, security, and trusted sharing of AI assets. The paper provides an overview of the XMANAI platform main functionalities, addressing the challenges faced during the development and presenting the evaluation framework to measure the performance of the delivered XAI solutions. It also demonstrates the benefits of the XMANAI approach in achieving transparency in manufacturing decision-making, fostering trust and collaboration between humans and machines, improving operational efficiency, and optimizing business value.

## Introduction

Explainable AI (XAI) models belong to the class of models that provide insights into how an AI system makes predictions and executes its actions. In recent times, these models have gained significant attention due to their transparency and interpretability in domains such as finance and healthcare. However, such models often fail to successfully address complex problems in highly dynamic environments with multiple parameters involved (Lampathaki et al., 2021). This poses a major challenge in using these models in industrial settings such as manufacturing. In those cases, achieving a balance between transparency and accuracy in XAI systems is crucial, as there is a trade-off between these aspects.

The XMANAI project (www.ai4manufacturing.eu) has defined a concrete strategy to address this trade-off and provide explainable yet performant solutions for the manufacturing domain. Several methods have been identified that lay an underlying foundation to building this strategy. It includes an efficient and trusted handling of data along with the possibility of creating pipelines for all XAI asset lifecycle management. Thereupon, the XMANAI platform as a prototypical solution is developed to realize and evaluate this strategy.

The primary goal of this paper is to introduce the approach used to build customizable XAI solutions for the manufacturing industries. The key factors considered in building this approach are security, interoperability, trusted sharing, transparency and asset lifecycle management. These can be mapped into three major dimensions to view the XMANAI platform, which are the Data, Services and AI models. The data dimension introduces the secure and efficient handling of all major XAI assets including datasets extracted from the manufacturing operations, AI pipelines, trained models and explainability results. The services dimension introduces the means to design, apply and manage XAI algorithms and the AI models' dimension gives a peek into the XAI model paradigm that addresses concrete manufacturing use cases.

The XMANAI platform is developed and deployed as a solution to specifically address the limitations of traditional AI models in the context of the manufacturing industry. One of the main challenges in applying AI in industrial settings is the lack of transparency and interpretability of black box models (Sofianidis et al., 2021). In this paper, we present how the XMANAI platform helps to overcome this challenge, as a secure and trustworthy environment where data scientists together with business users can create and customize interpretable AI solutions by combining XAI methods with domain knowledge. We provide a comprehensive overview of the platform's key features and capabilities, highlighting how it enables the collaborative construction of transparent AI models that can be understood by agents involved in the decision-making process. The challenges faced during the development of the platform are also discussed.

Finally, this paper presents an extensive evaluation framework by utilizing novel methodologies for assessing business value and evaluating the impact of AI systems. The extended 6P methodology and the use of Fuzzy Cognitive Maps provide valuable insights for measuring the effectiveness and added value of XAI in manufacturing. By sharing these methodologies, we aim to facilitate further research and implementation of XAI in industrial domains. We strive to empower decision-makers, data scientists, and business users in the manufacturing sector to make informed decisions, optimize business value, and establish trustworthy collaboration between humans and machines.

This paper is structured as follows: In Section 2, we provide an overview of the existing literature on Explainable AI and its applications in various domains. We identify the gaps in the current approaches when it comes to the specific challenges faced in the industrial sector. In Section 3, we present an overview of the XMANAI approach to the provision of explainable data, models and services. We demonstrate the XMANAI platform and its key features that enable the construction of transparent AI models for manufacturing applications. Section 4 discusses the validation of business values within the XMANAI pilot demonstrators, highlighting the extended 6P methodology and a novel method for assessing business added value using Fuzzy Cognitive Maps. Finally, in Section 5, we conclude the paper by summarizing the contributions and discussing future directions for research and implementation of XAI in manufacturing.

## Literature review

Explainable by design methods play a crucial role in enhancing transparency and interpretability in machine learning (ML) models (Lipton, 2018). Notable examples found in literature include linear and rule-based models, decision trees, k-nearest neighbors (KNN), or Bayesian models (Branco et al., 2023). Each method offers unique strengths and/or limitations, providing valuable insights into the decision-making process and AI interpretability.

Linear models provide interpretable outputs by combining input features linearly. They offer intuitive explanations of feature contributions, allowing for easy simulation by humans. However, their simplicity limits their ability to capture complex relationships present in certain datasets (Hastie et al., 2009). More complex rule-based models offer transparency through logical rules. However, as the number of rules increases, the model complexity grows, potentially leading to a loss of interpretability (Caruana et al., 2015). Fuzzy rule-based models enhance interpretability by incorporating fuzzy logic, providing more nuanced decision-making capabilities (Chimatapu et al., 2018).

Conversely, decision trees offer a graphical approach to explainability, dividing the data space based on decision rules. They provide clear visualizations and are applicable to both classification and regression tasks (Podgorelec et al., 2002). However, decision trees can become overly complex and prone to overfitting, leading to reduced generalization performance. Different algorithms offer varying splitting methods, branching strategies, and loss criteria, impacting their interpretability and accuracy (Breiman, 1984). KNN models provide simplicity and interpretability by classifying data based on the majority class among nearest neighbors, making them valuable for understanding predictions (Imandoust and Bolandraftar, 2013). However, KNN models are sensitive to the choice of distance metrics and the number of neighbors considered, hence may struggle with high-dimensional datasets.

Finally, and still explainable by design, Bayesian models establish probabilistic connections between features and outputs, capturing uncertainty through prior probabilities and likelihood functions. They offer inherent transparency, allowing the analysis of variable contributions through directed acyclic graphical models (Scanagatta et al., 2019). However, Bayesian models can be computationally intensive and require careful selection of prior probabilities and model assumptions (Betancourt, 2017). In summary, each explainable by design method has its unique characteristics and it is important to understand their strengths and limitations before selecting an appropriate approach for a specific application, and acknowledge that trade-offs must be made between interpretability, complexity, and computational demands to ensure transparency in machine learning models. However, when dealing with complex problems encountered in industrial settings, black box models are often preferred due to their efficiency.

*Post-hoc* explainability techniques play a crucial role in understanding the behavior of complex ML models after training (Guidotti et al., 2019). These techniques provide valuable insights into how these models arrive at their predictions. They can be broadly categorized as model-agnostic or model-specific, depending on their applicability to different types of models (Adadi and Berrada, 2018; Molnar, 2021). Model-agnostic techniques offer flexibility by separating the explanation technique from the ML model itself. They can be applied to any ML model, making them a versatile choice, particularly when comparing explanations across different black-box models. However, it's worth noting that model-agnostic methods may sacrifice efficiency or accuracy compared to model-specific techniques, as they do not leverage the specific features of each model (Ribeiro et al., 2016).

One model-agnostic technique is explanation by simplification as it involves using a surrogate interpretable model to approximate the predictions of the initial black-box model (Tritscher et al., 2020; Seddik et al., 2022). This surrogate model, designed to be explainable, helps understand the behavior of the initial ML model and facilitates comparisons. Nonetheless, the accuracy of this approximation may be limited (Ribeiro et al., 2016). Another well-known model-agnostic technique is explanation by feature relevance (Barredo Arrieta et al., 2020; Tritscher et al., 2023). Its goal is to measure the contribution of each input feature to the model's prediction. Various methods fall into this category, including Partial Dependence Plots (PDP), Individual Conditional Expectation (ICE), and SHapley Additive exPlanations (SHAP). They provide insights into the relationship between the target variable and individual features, enabling users to gain a deeper understanding of the model's behavior (Lundberg and Lee, 2017). Finally, visual explanation techniques are also model-agnostic and provide visually interpretable explanations of black-box model predictions (Barredo Arrieta et al., 2020). These techniques combine visualizations with other methods to enhance understanding and capture complex interactions among variables. Common visualizations used in these techniques include box plots, bar plots, heatmaps, and scatter plots, which help convey the learned patterns of the model (Molnar, 2021).

Model-specific techniques are tailored to explain specific models or categories of models. They rely on mathematical or statistical analyses specific to certain types of models to extract meaningful reasoning for predictions. For example, tree-based ensembles, Support Vector Machines (SVM), and Deep Learning (DL) models often require model-specific explainability techniques. Tree-based ensembles, such as random forests and gradient boosting, can be interpreted using techniques like explanation by simplification and explanation by feature relevance (Kuralenok et al., 2019; Lundberg et al., 2020). These methods provide justification for the predictions made by ensemble models. Explanation by simplification, feature attribution, and visualizations have been commonly used to shed light on SVM models and understand how they make decisions (Van Belle et al., 2016; Shakerin and Gupta, 2020). Similarly, deep learning models, including Deep Neural Networks (DNN), Convolutional Neural Networks (CNN), and Recurrent Neural Networks (RNN), require specialized explainability methods. Feature relevance, model simplification, and feature visualization techniques are often used to gain insights into these models and comprehend their decision-making processes (Samek et al., 2017).

### XAI in industry

Industry 4.0 has revolutionized manufacturing by introducing advanced technology to prioritize customer needs and customization. This has led to continuous improvements in quality and productivity. Smart manufacturing and smart factories, driven by intelligent systems, allow for flexibility in meeting varying product demands (Marques et al., 2018). Recent developments in IoT, Cyber Physical Production Systems (CPPS), and big data have further enhanced productivity, quality, and process monitoring in the industry, and AI has become increasingly important in the transition to the 5.0 paradigm, with more manufacturers integrating AI into their operations (Ogrezeanu et al., 2022).

Modern industry 5.0 settings rely on a high level of automation and collaboration between humans and machines. Throughout the manufacturing production line, various processes are continuously monitored using multiple sensors and controlled by actuators. Real-time analysis of these measurements involves the use of SVM (Doltsinis et al., 2020), random forest (Wang et al., 2018), CNN, or reinforcement learning (Kuhnle et al., 2021) models to evaluate the status of each sub-system involved in a specific process. Peres et al. (2020) identify data availability, data quality, cybersecurity and privacy preservation, and interpretability/explainability as key challenges to drive the industrial adoption of Industrial AI with interpretability tools as the main catalyzer. Hence, to effectively integrate AI solutions into such complex and dynamic environments, decision makers and operators need to monitor the process, understand the decisions made by AI components through explainability techniques, and intervene manually when necessary (Rožanec et al., 2022).

Research confirms that comprehending the inner workings of ML and deep learning models is crucial for data specialists and scientists but also for industrial experts. Much work has been recently developed in the field of XAI for industry, where explainable and interpretable methods have proven to be successful in many applications (Ahmed et al., 2022). Schlegel et al. (2019) and Bharti et al. (2020) are using XAI to interpret ML algorithms performing time-series analysis for demand planning and forecasting activities. Production management, such as the interpretation of the predictive analytics concerning defective products can also be found in literature (Kharal, 2020). DNN models have demonstrated strong performance in detecting defaults using computer vision, as they can identify anomalies that are difficult or impossible to detect with the naked eye (Wang et al., 2018). Explanations such as saliency maps provide visual insights that enable operators to effectively oversee quality inspection processes.

Predictive maintenance is another industrial application where XAI is being widely used. It involves real-time data collection to monitor the condition of devices and identify patterns that can aid in predicting and proactively anticipating malfunctions. Krishnamurthy et al. (2020) proposed an XAI framework for predictive maintenance in automotive applications, and Brito et al. (2022) employed anomaly detection and Shapley additive explanations (SHAPs) for interpreting their fault detection models. The study conducted by Chen and Lee (2020) illustrates the synthesis of a range of *post-hoc* explainability techniques to elucidate the inner workings of deep CNNs used for machine bearing fault diagnosis. These works present valid targeted solutions for specific manufacturing problems. However, a comprehensive approach enabling not a single solution but different AI explainability techniques, designed from the conceptualization phase addressing, security, interoperability, trusted sharing, transparency and asset lifecycle management is missing. One way to enhance explainability is to leverage multiple techniques that complement themselves to make the AI results more interpretable and transparent.

## The XMANAI approach—Explainable data-models-services

XMANAI set out to develop a holistic approach to develop robust and insightful XAI pipelines that can assist manufacturers in their everyday operations and decision-making processes, considering not only individual components of data, models and services but their interconnected value, through a collaborative platform in which different AI explainability techniques are enabled for a plethora of manufacturing applications.

### Data dimension

Having high-quality data is a requirement to create insightful AI pipelines, however the importance of having common underlying structure and semantics for these data is not always obvious. Indeed, even without a common data model, data scientists can explore, query and understand the available data, train and evaluate machine learning models.

Using a common data model during data ingestion has many advantages. It allows the stakeholders that have knowledge over the data, e.g., business users, to pass this information to the teams that will be responsible for data analysis. Semantics will thus stay with the data, ensuring an understanding for the data scientists even in the first steps of exploration. Spotting anomalies in the data, developing an intuition as to the expected distributions, creating meaningful visualizations, anticipating required transformations and thinking of potentially useful combinations, are among the processes that become easier based on this availability of data insights, thus accelerating everyday operations of data scientists when it comes to exploratory data analysis.

Going a step further, a data model is not only related to the input data but can be also leveraged for derivative data generated during data pre-processing, feature engineering and finally results extraction from the AI models and analysis processes. In this way, data across the AI pipelines are enriched with semantics conveying to stakeholders what the data are about and how they can/should be handled. Having data that essentially “carry their meaning” directly contributes to the overall explainability of the XMANAI AI pipelines. A common data model also allows the implementation of more advanced data validation rules, guarantees data integrity, and facilitates deployment. Furthermore, data manipulation operations can be enabled or disabled depending on the data types/units of the data being used.

Finally, having this common understanding of the data being used and enforcing certain actions, rules, quality, and security tests based on commonly agreed upon structures, does not only facilitate the operations of each stakeholder role in XMANAI, but provides the foundations for a more productive collaboration among business users, data scientists and data engineers.

#### XMANAI graph data model for the manufacturing domain

After studying some of the existing and commonly used domain vocabularies, including ISO 10303,^1^ ISO 15926 (Klüwer et al., 2008), X3D ontology (Brutzman and Flotyński, 2020), the XMANAI graph data model has been defined re-using their core concepts for manufacturing, and is used in various steps of the XAI pipelines to add semantics to the data. The XMANAI Data Model describes the nodes, their properties and relationships. Its current version comprises a total of 48 concepts spanning across different aspects, processes and entities of the manufacturing domain, including 3D Representation, Machine Monitoring, Market (Product and Customer), Production, Quality Control, and KPI, Sensor data, almost 300 properties and more than 700 relationships.

Indicative examples of the data model's usage include leveraging the defined types and measurement units to help detect issues in the input data in a timely manner before these are propagated in training processes, using the available semantics to enable/disable feature creation and other data manipulation functionalities accordingly, making feature importance insights easier to comprehend by keeping the link to the overall structure and relationships among data. Integrating the data model in the AI lifecycle is also expected to contribute toward collaboration, as all stakeholders will have a common view and understanding on the data and models that drive the AI pipelines.

**Figure 1:**
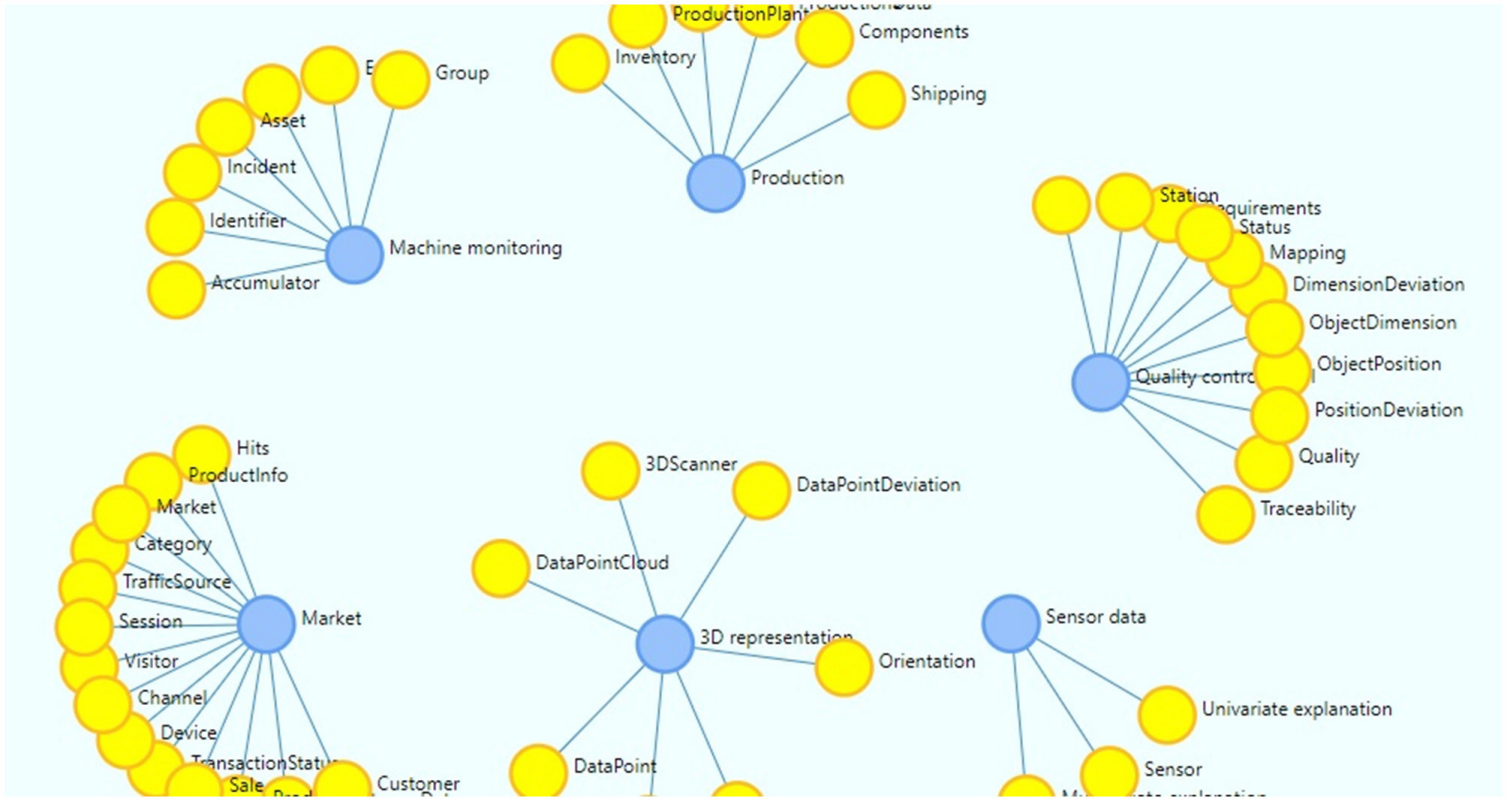
XMANAI data model overview.

The knowledge graph manager is a component designed to provide a clear visualization on how each node correlates with another and get a better understanding of the model as well as to get assistance on data mapping activities. Being the data model represented as a live graph, the Knowledge Graph Manager also provides the capability to expand and update the model with new concepts or properties, enabling the initial deployed data model to evolve through extension of the concepts, properties and relationships to the dynamic nature of the manufacturing domain. An overview of the XMANAI Data Model is displayed in Figure 1.

#### XMANAI secure and efficient data management and sharing services

Manufacturing industries generate large volumes of unstructured and sensitive data during their daily operations. This data belongs to various formats and configurations. To maximize effective utilization of this data, several methods have been adopted to build a secure and efficient data management and sharing system. This system manages processes to import, store, transform and track data and brings transparency, security and explainability to the data. In XMANAI, these methods adhere to not just data generated in industrial surroundings, but digital assets which includes processed/transformed data, trained models, explanations, pipelines, scripts, results, predictions and analytical reports of XAI pipelines. The methods identified to build the above-mentioned data management and sharing system are explained below.

##### Industrial asset ingestion and standardization

Ultimately the practical benefit of the XMANAI platform for businesses in manufacturing should be an increase of one or multiple performance indicators, such as product quality or sales revenue, yielded by applying XAI methods on their own data. This leads to a context in which access to the most recent data may directly translate into increased performance of the company. Thus, an efficiently operating ingestion procedure to feed the data into the ecosystem of the analytics platform while minimizing information overflow is desired (Gorawski and Gorawska, 2014). Due to the lack of cohesion in industrial data, the data ingestion system must provide the flexibility to integrate heterogeneous data belonging to various formats and schemas.

As the interoperability of data significantly affects how efficiently it can be used (Gal and Rubinfeld, 2019), its standardization plays a major role for data-centered applications. As explained previously, XMANAI promotes the usage of a common data model to facilitate operations like the merging of different datasets, which is the foundation for actions like the updating of data.

With the goal of providing a standardized data ingestion, which is flexible, efficient and transparent, XMANAI proposes a solution involving multiple components with different tasks: The API data harvester, which is an ETL (Extract Transform Load) tool that connects to data providing APIs and offers a graphical user interface, allowing the configuration and automation of harvesting processes, through which the data gets pulled from the desired source, transformed and ingested into the XMANAI ecosystem. For data to be ingested, and stored in binary format, the file data harvester can be utilized. It will extract the data, transform it and make it usable for further processes within the XMANAI ecosystem. The registry is storing all metadata associated with an asset and providing it to other services like the file data manager for transparent and efficient data management as well as for the uploading of data in the form of binary files. Both file data harvester and API data harvester offer a configurable data conversion tool, allowing for an individualized transformation of the data into the XMANAI data model.

##### Industrial assets provenance

To be compliant in a world, where data security plays an increasingly important role, data-intensive projects have to make sure that they have control over their information streams, involving which changes were introduced to the data, when and by whom (Colin et al., 2012). Provenance records are metadata information of an asset which includes *why, where, how, when* and *by whom* an asset was created, updated, read or deleted. These records also store information about actions performed on assets when multiple versions of the same asset exist in the database. An efficient version control mechanism of assets in the database goes hand-in-hand with generating provenance information and scalability of this version control is a major challenge when it comes to storing large volumes of industrial data.

In the context of XMANAI, assets store with version control is the centralized database that stores all data assets and includes the mechanism to store and retrieve multiple versions of an asset. The provenance engine documents all types of CRUD (Create Read Update Delete) operations being performed on versions of data assets, including the timestamp (when) and actor (who). The respective metadata is being stored in RDF format, as triplets according to the W3C-PROV Ontology (W3C, 2013) yielding a continuously expanding knowledge-graph and providing maximum transparency over the lineage of an asset.

##### Security, privacy, and trust considerations

In XMANAI, our focus is on the security of the data in motion, data access control, data anonymization and identity and authorization management. Secure transportation of data is handled by the transfer protocols that exist in various layers of the TCP/IP model. To guarantee restrictive access to the critical and sensitive data assets, Access Control Mechanisms (ACMs) cover all the authentication and authorization aspects of industrial data. Identity and authorization management constitutes of the user with a digital identity, the Identity Provider (IdP) who generates and maintains the digital identity and the Service Provider who has the service that utilizes IdP for the verification.

XMANAI utilizes a policy engine to regulate the access to data assets with the help of access control decisions for each access request. Along with it, the *Policy editor* presents a user interface through which the asset owner can perform all access policy lifecycle management activities. Identity and authorization management performs the registration, verification, and authentication of users and serves as the identity provider of the XMANAI platform.

##### Industrial assets sharing and IPR handling

Industrial assets include digital assets generated in industries or derived from it. Even though data marketplaces are not a new term in the current technological landscape, the fear surrounding them is about losing negotiation power or unknowingly giving away sensitive data. Another critical aspect for such marketplaces is efficient handling of data breaches. Intellectual property rights (IPRs) are licenses that formulate decisions related to industrial data access and data sharing. It helps to safeguard the rights of the data owners over their property. As aggregation of multiple datasets is common in building XAI pipelines, if they are licensed under different licenses containing several terms and conditions, IPR conflict resolution is a challenge that needs attention.

XMANAI proposes a XAI marketplace for assets sharing that includes a Metadata Manager and a Contract Manager. Metadata manager stores and maintains the metadata of assets along with the licensing details such as license type and IPR owner. Contract manager controls asset sharing between organizations and its users. It allows the creation of smart contracts, terms enforcement and other interaction between users.

### Services dimension

To realize its goal of bringing explainable AI to the manufacturing domain in an effective manner, XMANAI offers the means to design, apply, and manage different XAI algorithms to a plethora of different manufacturing applications. The provision of robust and insightful XAI algorithms that can assist manufacturers in their decision-making processes, goes well beyond ML models' training by data scientists working in isolation. The next two sections present the services that have been already delivered by XMANAI to handle the complete lifecycle of these algorithms through XAI pipelines, and the role visualization and collaboration play in this regard to help fuse XAI insights into the manufacturing operations.

#### XAI algorithm lifecycle management

An important aspect of developing machine learning solutions is that training and applying algorithms is only part of, and not the complete process, as well as the fact that the delivery of the trained algorithm is not the final step. Starting from data understanding through exploration, moving to model training and evaluation, and then to production application and monitoring, whilst foreseeing feedback loops and risk management, the lifecycle of the ML algorithm is in fact the lifecycle of the pipeline through which it is created. These pipelines in XMANAI build upon the industrial data ingestion and the common domain model presented in the previous section, and a set of interconnected, yet modular, services is provided for their design, management and deployment. Establishing the processes to fuse explainability across the complete pipelines' lifecycle has been a core goal in the design of the services and the components that offer them, namely:

(i) The XMANAI interactive data exploration and experimentation tool (IEET) that covers the essential role of experimentation in data science. A typical ML pipeline consists of a sequence of steps, like data preprocessing, model selection, hyperparameters tuning, and XAI techniques. For determining the appropriate approach in each step, data scientists typically need to familiarize themselves with the dataset's characteristics and apply multiple try-error iterations. IEET helps data scientists gain valuable insights to make informed decisions for subsequent pipeline steps. IEET follows the typical Notebook paradigm, offering a set of predefined environments and templates which can be optionally used to accelerate the experimentation process.

(ii) The XMANAI data preparation engine, which allows users to define and configure various data processing steps, spanning from filters and dataset combinations to mathematical operations, to information extraction and feature engineering. The core scope of this component is to apply the necessary transformations to render the data suitable for visualization and model training purposes.

Model selection, configuration, training and application functionalities are provided through the (iii) XAI model engineering engine, which allows training a model from scratch or re-training it from a previously completed experiment, configuring its hyperparameters and validation strategy and visualizing its training metadata. The XAI Model Explanations Engine oversees the configuration and application of the appropriate explainability methods, as well as the visualization of the generated explanations from a scientific perspective, while also offering simplified versions for less technical users.

(iv) The XMANAI experiment tracking engine that is based on MLFlow and enables logging of experiments' metadata (performance metrics, corresponding hyperparameters and other artifacts) and performing comparisons in the performance of various trained models across selected dimensions.

(v) The pipeline designer, which lies at the heart of the XAI pipelines, as it enables and coordinates the collaboration of different stakeholders (business experts, data scientists and data engineers) and team members across the various steps of the pipelines. Through the pipeline designer, users can graphically select, configure and link steps from the numerous services (data preparation, feature generation, prediction model training, explainer model training, explanation request, etc.) to create a chain of functionalities up to storing derivative assets, including for prediction results and generated explanations. Discussion functionalities are offered at the pipeline and at the step level fostering collaboration among stakeholders.

(vi) The execution and orchestration engine, for scheduling and executing XAI pipelines based on the specified execution modality, cloud or on-premise, and predefined environment. It generates the necessary information to create the actual pipeline, utilizing the configuration provided by the Pipeline Designer.

(vii) The pipeline serving and monitoring engine that takes care of deploying the pipelines in a production-ready manner with appropriate settings. It also monitors the performance of these pipelines, providing valuable insights regarding their operation.

#### Visualization—Collaboration

Visualization plays a crucial role in the complete lifecycle of XAI pipelines, providing a common language and visual representation not only of data but also ML models. Visualizations are effective tools for communicating and presenting findings to business users, allowing often complex information to be conveyed in a clear and intuitive manner. Therefore, visualizations targeting the end users' needs are provided to make it easier for non-technical audiences to understand and make informed decisions based on the insights generated by AI models.

As explained, diverse visualization needs have been addressed by the delivered interfaces to help untap the XAI potential in the manufacturing domain. An additional benefit of this facilitated shared understanding is that it enables effective communication and collaboration between team members with different backgrounds and expertise. Data scientists and data engineers are encouraged, through the provided components' interfaces, to collaboratively explore and understand data, detect data quality issues, and make informed decisions about data preprocessing and feature engineering processes. By providing visualizations across all phases of an XAI pipeline's development process, targeting different needs of all stakeholders, fruitful discussions are promoted leading to more efficient XAI pipelines, from design to production. Business users can provide domain expertise and insights that help interpret these visualizations, leading to more meaningful and actionable interpretations. Collaboratively creating and refining visualizations allows data scientists, data engineers, and business users to align on the key messages and make data-driven decisions together. Visualizations allow business users to explore the data and models on their own, fostering engagement and participation in the decision-making process.

### AI models dimension

#### XMANAI hybrid XAI model paradigm – coupling “black-box” ML models to *post-hoc* explanations

Modern industries present dynamic environments where high-dimensional, non-linear problems often need to be addressed. Interpretable by design models such as linear models and Decision Trees often fail to efficiently address such tasks and are usually outperformed by complex algorithms such as Neural Networks and ensemble models. The XMANAI approach to overcome the “performance vs. interpretability” trade-off is based on the idea of coupling complex AI models to suitable XAI components that explain the model's decisions. Since the AI model is linked to its (one or more) explainer throughout its lifecycle, the combination of the two can be viewed as a Hybrid XAI model, consisting of the original AI algorithm with an additional explainability layer. Under this approach, “black box” AI models are transformed into “glass box” solutions for the manufacturing domain, offering to stakeholders the opportunity for transparent solutions that still maintain state-of-the art performance.

#### XAI models catalog

The XMANAI XAI model paradigm is applied to address concrete use cases in the manufacturing domain put forth by the XMANAI demonstrators, pertaining to process optimization, demand forecasting, anomaly detection and semi-autonomous planning. The proposed set of XAI models are the first to populate the XMANAI Models Catalog and available on the XMANAI platform. The design and development of XAI solutions for each case, comes as the result of collaborative work between XMANAI data scientists and business users dedicated to:

- Understanding the specific details and context of each use case, as well as the available data sources to address it.
- Defining the objectives/targets that each AI solution is expected to achieve, as well as the confidence levels of acceptable performance based on the company's needs.
- Identifying the specific needs for explainability to be fulfilled, i.e., which aspects regarding the response of the AI solution should be explained in detail to the end user.

Following this meticulous mapping of the use case and its requirements, XMANAI scientists are able to select an appropriate ML model to address its solution, while the corresponding XAI tool is selected based on the ability to provide explanations that meet the end user's needs. For example, SHAP values are employed in cases where feature attributions and interactions should be explained both at the global and at the instance level, also providing information on how feature values (high-low) affect the model's predictions. On the other hand, Permutation feature importance (Brieman, 2001) or a Decision Tree surrogate may be most suitable in cases where the interest is in explaining the overall behavior of the model at the global level.

#### Customization of XAI solutions

The direct outputs of XAI tools, however, are not always easy to conceive by the various business users, extending from Sales executives to machine operators. This has been verified within the XMANAI project by means of Explainability workshops and questionnaires targeting the business users and their specific needs for explanations. On the other hand, data scientists and data engineers lack the necessary domain expertise to fully interpret the AI models' explanations in the specific context of the application domain. Here is where the importance of XMANAI services comes into play, offering the means to data scientists and engineers to collaborate with business users over the design and management of custom XAI solutions. Building on the explainability outcomes in a way that incorporates domain knowledge and intuition, interpretable AI systems are created that provide useful insights to the end users. These solutions, enriched with text/alarm messages to the operator in natural language, have already been delivered into dedicated manufacturing applications, tailored to the needs of the XMANAI demonstrators. The delivered XAI solutions continue to evolve through constant feedback from the business users, until the end of the XMANAI project.

#### XAI model guard

The XAI Model Guard is implementing the XMANAI AI Security framework, delivering a set of functionalities that enable the safeguarding of the security and integrity aspects of the produced AI models. The particular framework is built around the following main axes: (a) the identification of potential adversarial attacks against the produced AI models, (b) the assessment of the risks associated with such attacks and finally (c) the detailed reporting of the findings to the user for them to take the necessary corrective actions if needed, based on his/her expertise on the analyzed ML model. In terms of adversarial attacks, the XAI Model Guard supports the assessment of the risks associated with the following attack types: (i) Pre-training (poisoning) attacks in which adversaries attempt to introduce adversarial data points to significantly deteriorate the performance of the model and its ability to classify. (ii) Post-training (evasion) attacks in which adversaries attempt to modify the input data so that the AI model cannot identify or deliberately miss-classify specific inputs. (iii) Backdoor attacks in which adversaries attempt to alter and control the behavior of the model for a specific input.

## The XMANAI platform

### Introduction to the XMANAI platform

**Figure 2:**
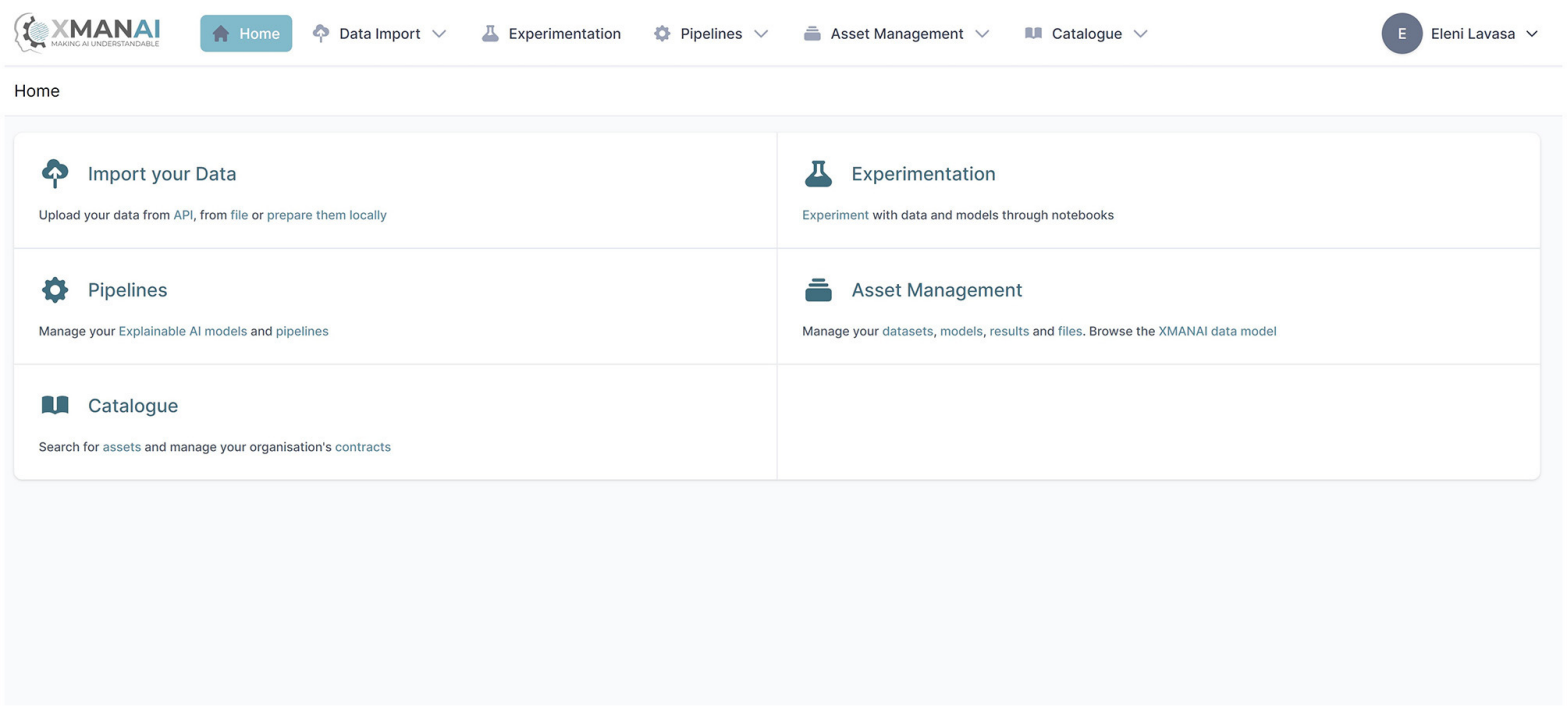
XMANAI platform menu page.

The current section aims to introduce the novel XMANAI Explainable AI platform (https://iam.ai4manufacturing.eu), putting together the methods and components introduced in the previous sections. The platform utilizes explainable AI models to instill trust, enhance human cognition, and effectively address real-world manufacturing problems by providing value-based explanations. The XMANAI platform empowers manufacturing stakeholders to solve specific manufacturing problems in a trustworthy manner, utilizing explainable AI models that offer easily interpretable value-based explanations for humans. At its core, the platform offers a catalog of hybrid and graph AI models that serve as reusable baseline models for addressing various manufacturing problems or as trained models fine-tuned for specific issues. These models have already been validated through four core use cases in the automotive, white goods, machinery, and metrology industries, utilizing innovative manufacturing applications and services. The XMANAI platform manages the entire lifecycle of AI assets, including data uploading, exploration, preparation, sharing, analysis, as well as the design and execution of AI pipelines, accompanied by value-based explanations and visualizations. The integrated XMANAI Explainable AI platform offers state-of-the-art data handling, data manipulation, and AI technologies and functionalities. A screenshot of the platform's menu appearing to authorized users is presented in Figure 2.

### The XMANAI platform architecture

The platform architecture is designed based on the principles of modular services and comprises of three core tiers, namely: (a) the XMANAI Cloud infrastructure which constitutes the core part of the platform and represents the centralized cloud instance of the XMANAI Platform, (b) the XMANAI On Premise Environments which represent the parts of the XMANAI Platform that can be hosted and executed in a private cloud instance of a stakeholder and (c) the XMANAI Manufacturing Apps Portfolio which is composed of AI manufacturing intelligence solutions that are effectively solving specific manufacturing problems. The XMANAI Cloud infrastructure comprises of (a) the Core AI Management Platform that provides all the offerings and functionalities of the platform and is responsible for the design of the data and/or AI pipelines and the orchestration of their execution; and (b) the Secure Execution Clusters (SEC) which constitute the isolated per stakeholder organization spaces which are triggered/spawn on demand by the Core AI Management Platform, for executing data and/or AI pipelines. The XMANAI on premise environments facilitate the execution of the platform's functionalities on the stakeholders' environments based on the instructions that are provided by the XMANAI Cloud infrastructure in accordance with the preferences of the stakeholder.

**Figure 3:**
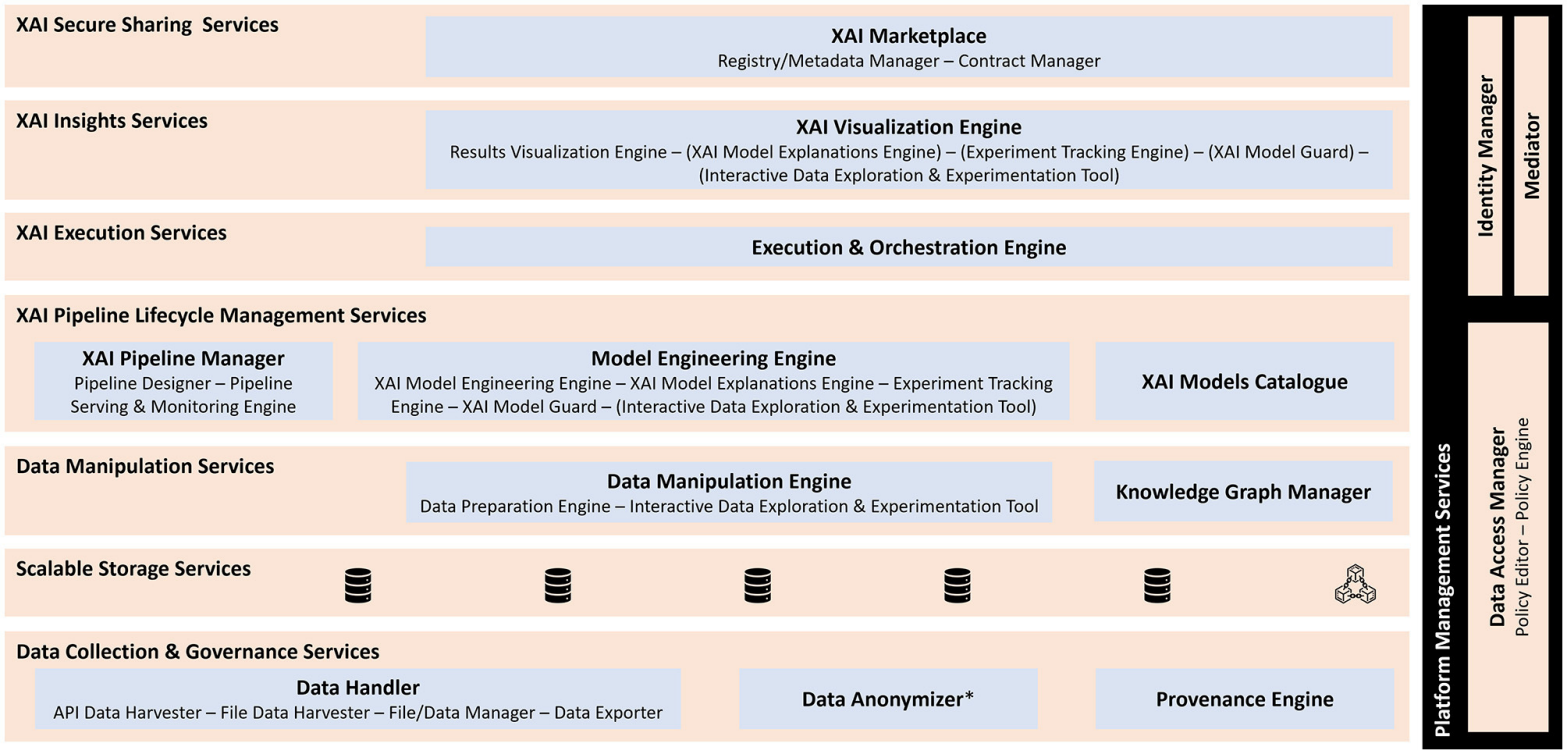
XMANAI Reference Architecture-Services Bundles Perspective. The star symbol (^*^) indicates that data anonymizer is based on a standalone tool (AMNESIA) developed by ATHENA RC.

The XMANAI platform's services are developed and integrated into eight distinct bundles, ensuring seamless integration through well-defined interfaces. These bundles facilitate intercommunication among the services and contribute to the overall functionality of the XMANAI platform as illustrated in Figure 3 and include the:

I. Data collection and governance services bundle, residing on both the XMANAI Cloud infrastructure and the XMANAI On-Premise Environments. Its primary responsibility is to ensure consistent and well-managed data collection by configuring and executing appropriate data handling processes. This bundle securely and reliably collects data assets and incorporates a provenance mechanism to track their lifecycle.
II. Scalable storage services bundle, residing on both the XMANAI Cloud infrastructure and the XMANAI On-Premise Environments. It facilitates the persistence of platform assets based on their types and storage locations (either centralized in the XMANAI cloud or on-premise, depending on the installation). Additionally, it provides metadata indexing to optimize query performance and enhance data discoverability.
III. Data manipulation services bundle, residing on both the XMANAI Cloud infrastructure and the XMANAI On-Premise Environments. Its core functionalities include data explainability and feature engineering. This bundle enables the derivation and harmonization of knowledge from available data based on the XMANAI data model. It also prepares the data for ML/DL applications, allowing its usage in training XAI models and executing XAI pipelines.
IV. XAI lifecycle management services bundle, residing only in the XMANAI Cloud Infrastructure. It manages XAI pipelines, encompassing collaborative design, validation, and handling of the pipelines. This bundle integrates various functionalities such as data preparation, model engineering, and explainability, as well as training, explanation generation, management, tracking, and evaluation of XAI models, considering performance and security aspects.
V. XAI execution services bundle, residing on both the XMANAI Cloud infrastructure and the XMANAI On-Premise Environments. It is responsible for executing XAI model/pipeline experiments during the experimentation phase and deploying XAI pipelines in the production phase based on user-defined schedules. This bundle monitors and tracks the execution status in the Secure Execution Clusters and/or the On-Premise Environments, ensuring the storage of model/pipeline results and associated metrics.
VI. XAI insight services bundle, residing on both the XMANAI Cloud infrastructure and the XMANAI On-Premise Environments. It facilitates collaboration between business experts and data scientists and supports gaining insights throughout different phases of extracting manufacturing intelligence. This bundle incorporates the XAI Visualization Engine, which offers novel dashboards and diagrams to visually represent data, XAI model results, explanations, and insights, thereby supporting the entire experimentation process.
VII. Secure asset sharing services bundle, residing only in the XMANAI Cloud Infrastructure. It enables cataloging and trusted sharing of data and AI models across various manufacturing organizations and/or users. These functionalities are provided through the XAI Marketplace.
VIII. Platform management services bundle, residing only in the XMANAI Cloud Infrastructure. Its responsibilities include access control functionalities for data assets based on providers' preferences, centralized user management, and authentication mechanisms for the platform.

## Validation of business value in XMANAI

### XMANAI pilot demonstrators

The XMANAI project is dealing with a set of underlying challenges and concerns that are critical for the industrial sector nowadays, such as:

- The need to reduce the maintenance costs.
- The need to reduce unplanned machinery downtime.
- The need to improve the Quality Control.
- The need to increase the production throughput.

**Figure 4:**
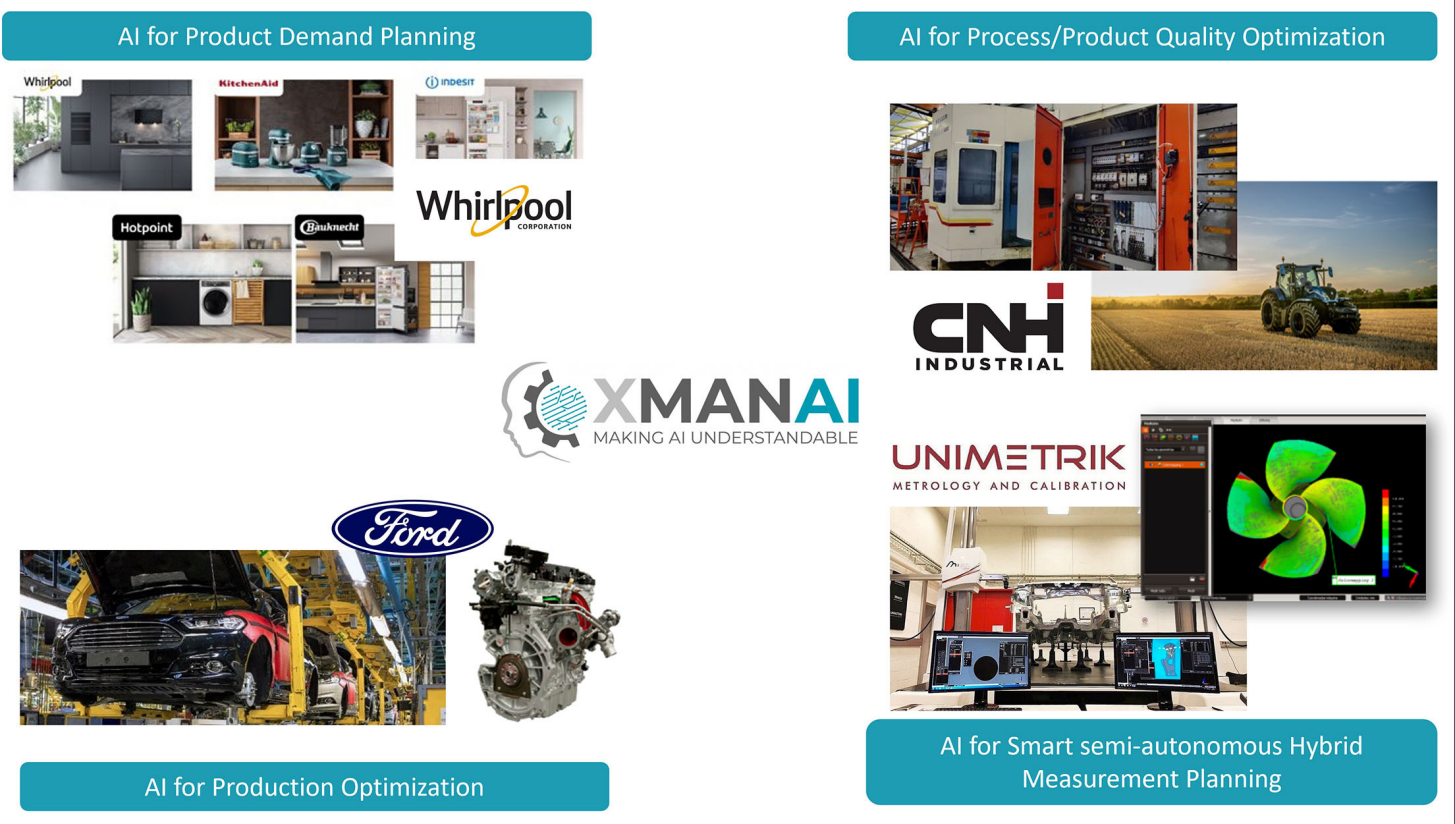
Demonstrators in XMANAI project that will test and validate the XMANAI solution.

XMANAI aims at bringing the Explainable AI benefits in different demonstrator cases addressing the above manufacturing challenges (Figure 4). XMANAI partners have applied the proposed approach to four industrial pilot demonstrators, enabling testing and validation of the solutions in real-life settings. As presented in the next sections, apart from the platform, custom manufacturing applications are developed for the companies' on-premise environments.

#### Demonstrator I. FORD

The Ford manufacturing application focuses on supporting machine operators and professionals in production facilities through two key use cases. The first use case involves providing a holistic overview of the production process, incorporating a joint representation of production line data and AI-driven product data predictions. It also includes an anomaly detection system and workload simulations based on hypothetical input scenarios. The application aims to alert users to unwanted scenarios and help them better understand the production process.

**Figure 5:**
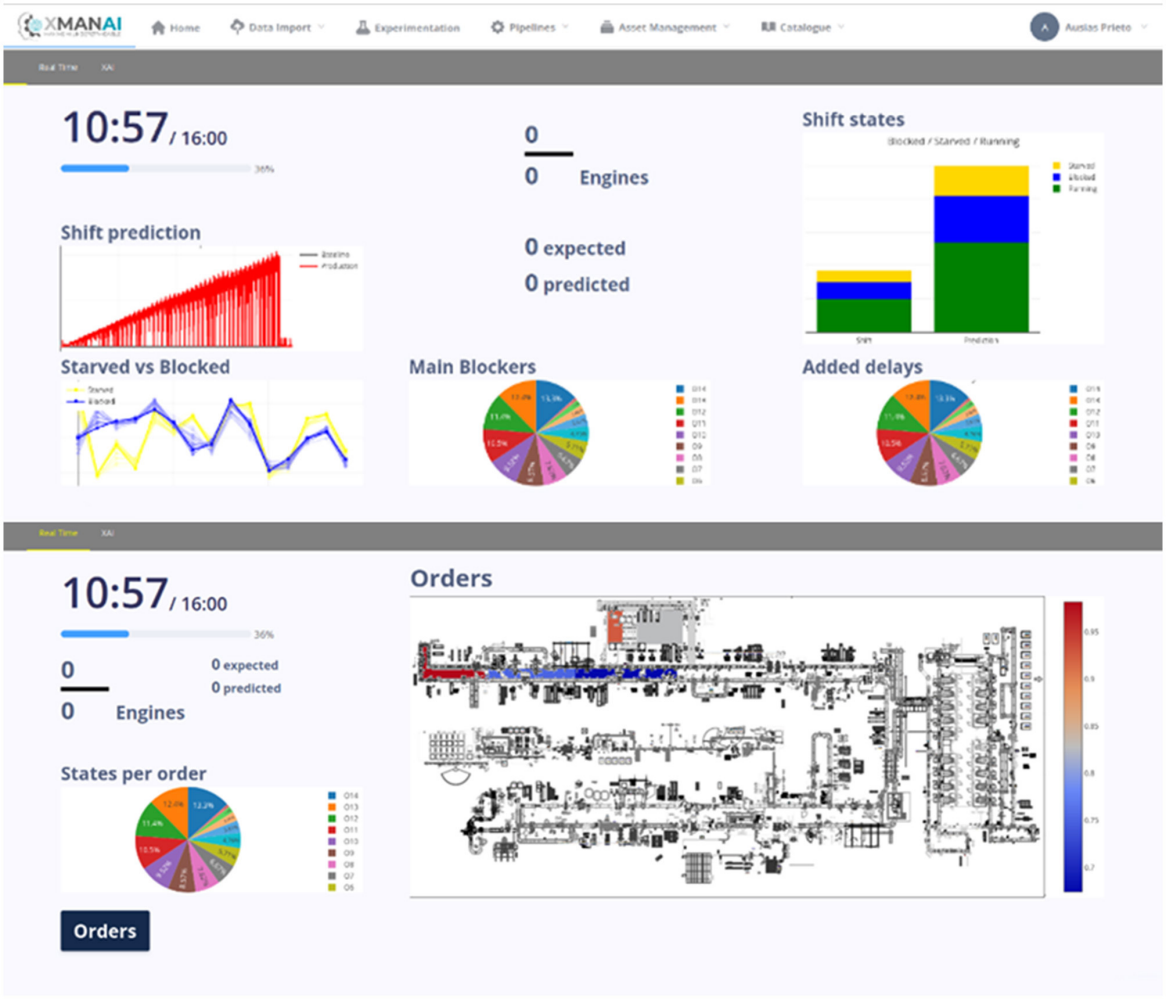
Indicative screenshots-FORD manufacturing application.

The second use case of the Ford application is Automated Production Planning. It assists in generating planning constraints, daily production planning, and sequencing, as well as monthly production planning. The system automatically retrieves data from the production line to provide a real-time status of operations and process cycle times. Additionally, it identifies parts of the production line that may affect predicted output, aiding in identifying root causes of problems when the predicted output deviates from the expected (planned) output. A collage of indicative screenshots of the application (alpha version) are presented in Figure 5.

#### Demonstrator II. WHIRLPOOL

**Figure 6:**
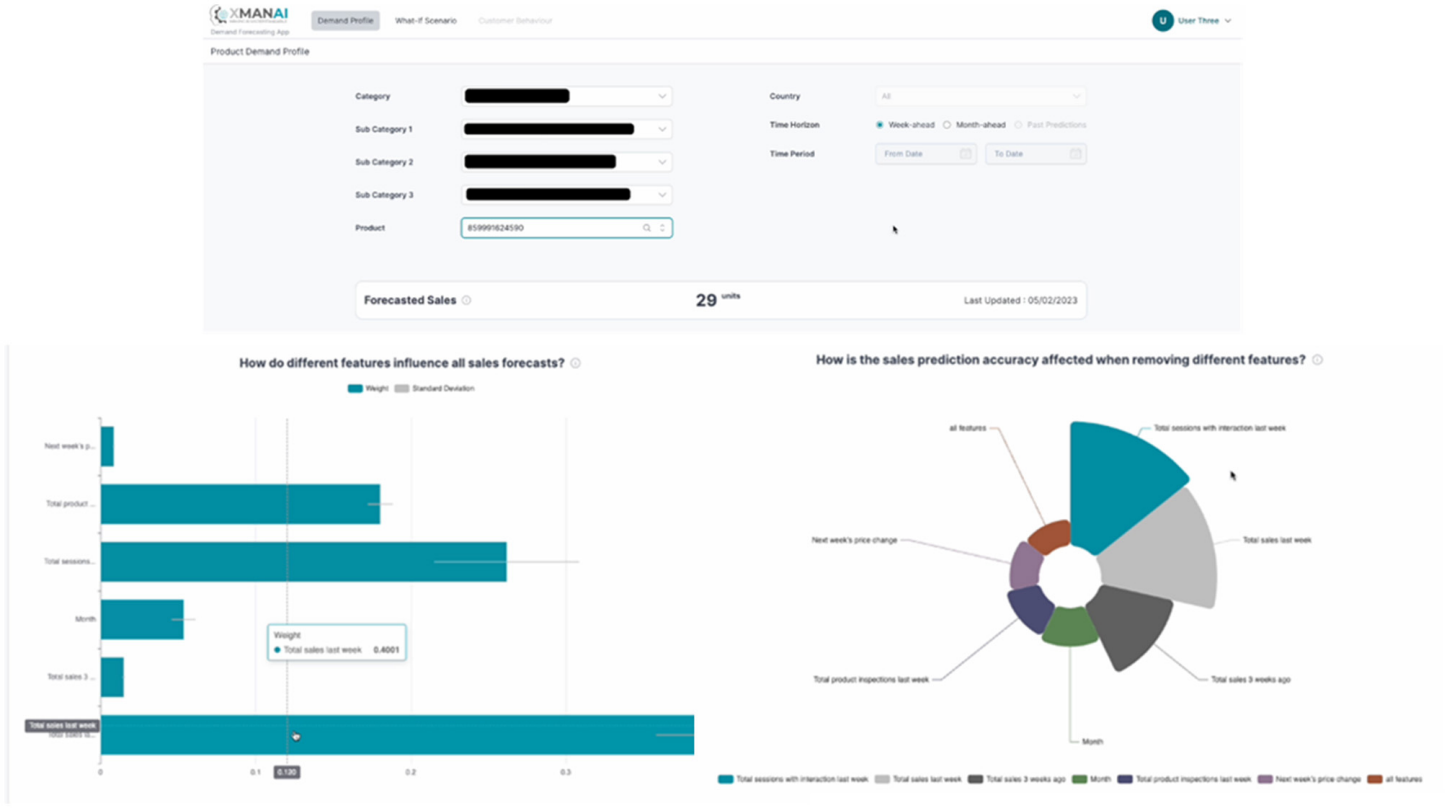
Indicative screenshots-WHIRLPOOL manufacturing application.

The Whirlpool manufacturing application is designed to enhance the decision-making process for two key stakeholders: the Central Demand Planning team and the D2C Marketing and Sales team. This application plays a crucial role in the overall demand forecasting process, generating the Operational Demand Plan for Whirlpool EMEA factories and markets. Indicative screenshots of the application (alpha version) are presented in Figure 6.

#### Demonstrator III. CNH

**Figure 7:**
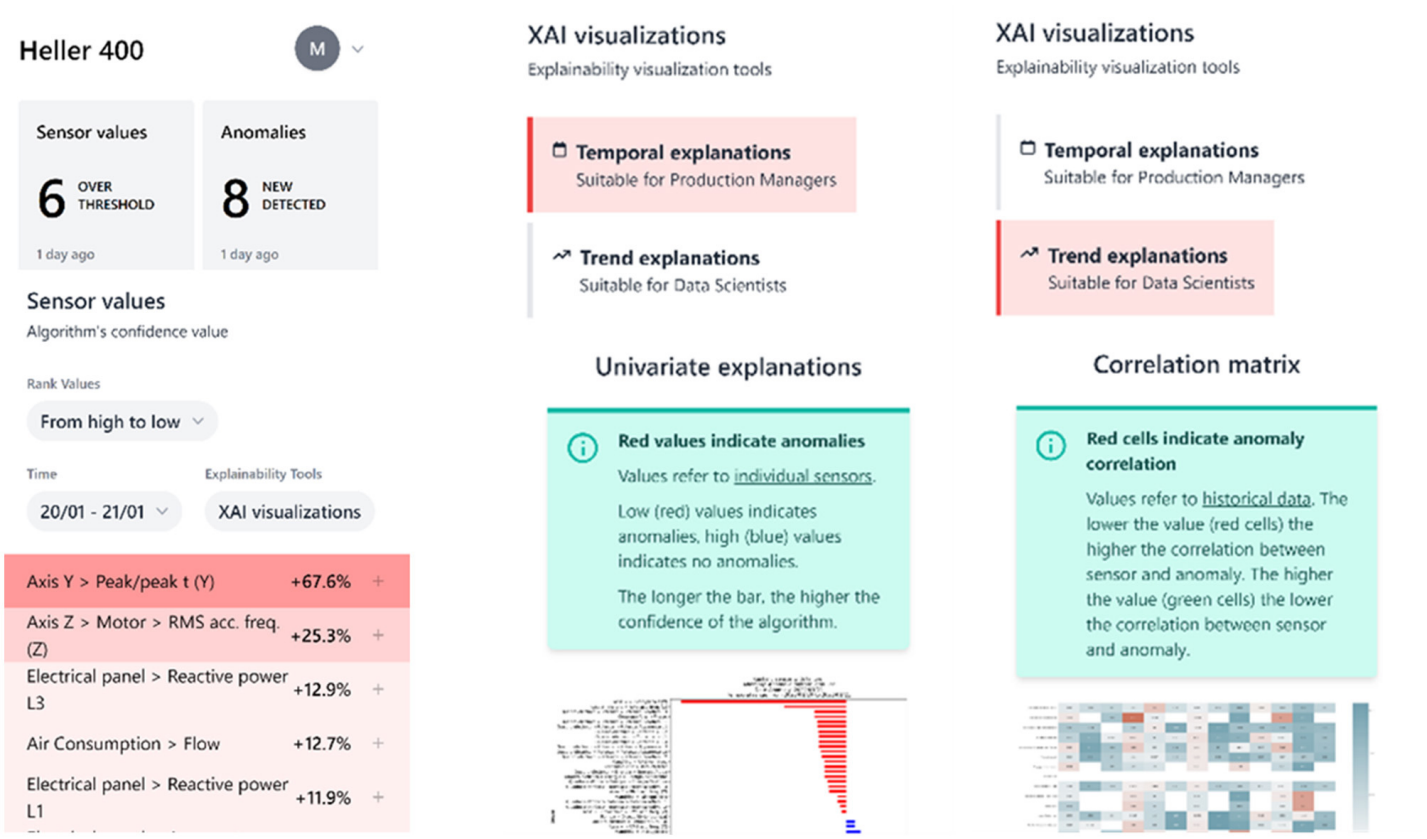
Indicative screenshots-CNH mobile manufacturing application.

The CNH manufacturing application aims to support machine operators in CNH production facilities when dealing with unplanned stoppages. By connecting the production line with an intelligent and explainable system, the application significantly reduces time and costs related to maintenance and production stoppages. The application addresses two primary use cases: anomaly detection, which helps identify unusual events or issues, and manage/forecast stoppages, allowing for better planning and mitigation of potential disruptions. Indicative screenshots of the application (mobile, alpha version) are presented in Figure 7.

#### Demonstrator IV. UNIMETRIK

The UNIMETRIK manufacturing application targets metrology technicians in the areas of Process Optimization and Semi-Autonomous Planning. This application encompasses two key use cases. The first use case involves Measurement Plan Optimization, assisting junior metrologists in their preliminary study of the part to be measured. It accelerates the process, minimizes costs, and ensures result consistency.

**Figure 8:**
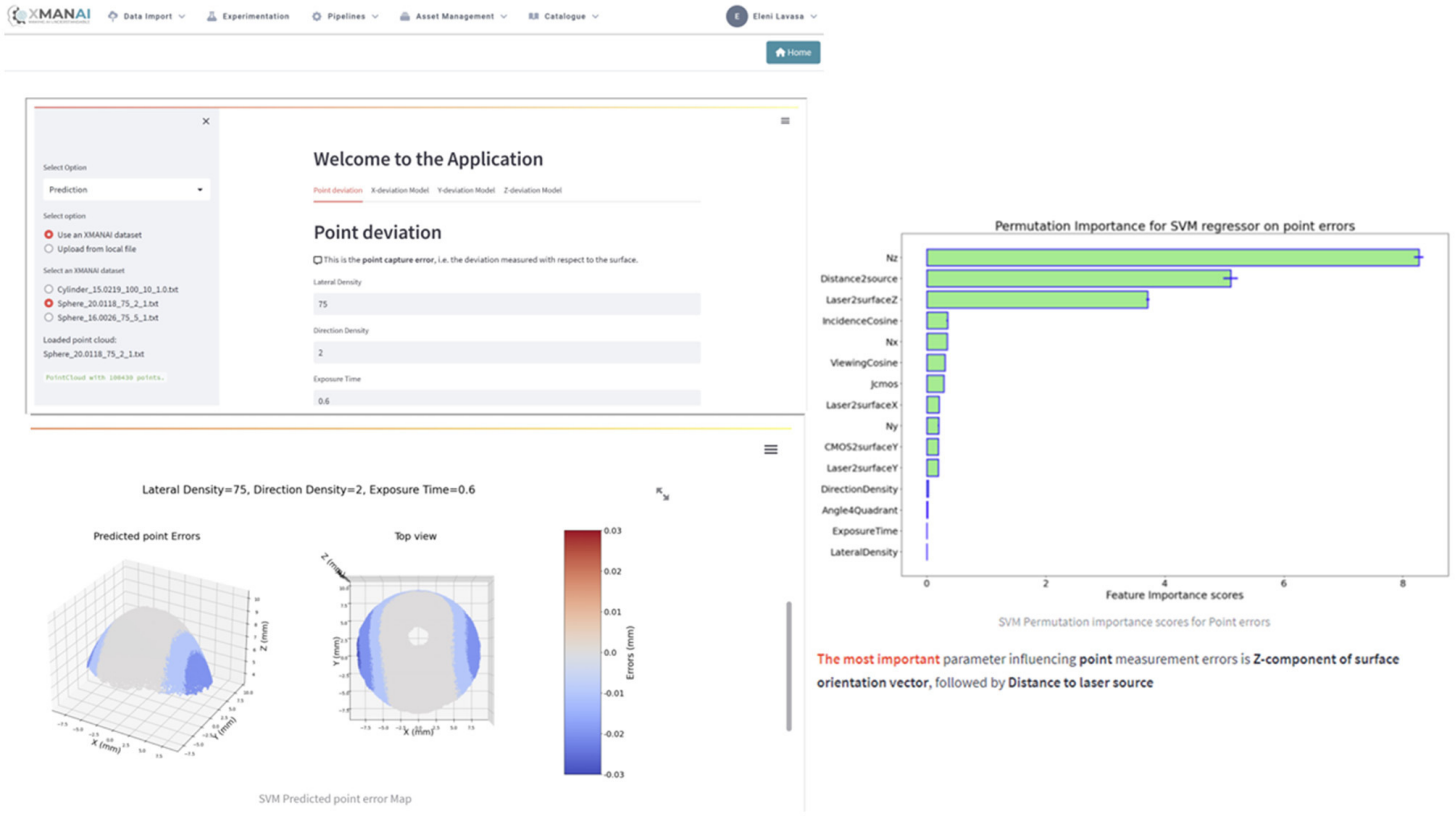
Indicative screenshots-UNIMETRIK manufacturing application.

The second use case focuses on Point Cloud Optimization, aiming to maximize measurement accuracy and minimize execution time while maintaining result consistency. The UNIMETRIK application empowers metrology technicians with advanced tools and capabilities to improve the efficiency and effectiveness of their work. Indicative screenshots of the application (alpha version) are presented in Figure 8.

### The extended 6p methodology

**Figure 9:**
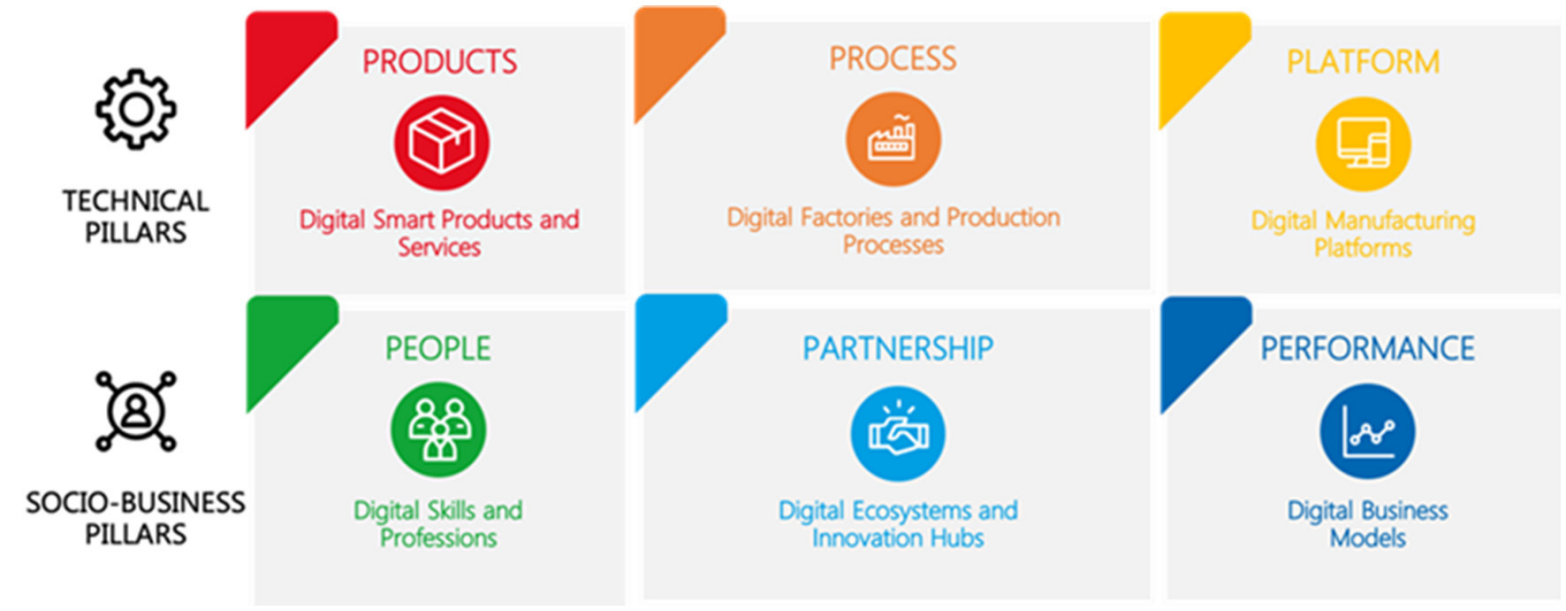
6Ps digital transformation tool-the six pillars.

The XMANAI project applies the 6P Methodology evaluation framework to assess the impact of Explainable AI (XAI) on the four demonstrators. This framework is built upon the existing 6P Migration Methodology, originally developed by POLIMI and inherited from the EU H2020′s AI REGIO Project. The primary goal of the 6P Migration Methodology is to aid manufacturing enterprises in their digital transformation journey by analyzing the six key pillars that characterize the production process, including both technical and socio-business dimensions (Figure 9).

In contrast to the original five-step 6P Migration Methodology, the XMANAI evaluation framework focuses solely on two crucial steps:

1. Identification of the AS-IS profile of the manufacturing enterprise: This step involves analyzing the manufacturing enterprise's strategy, competitive strengths, weaknesses, etc. The current profile is mapped into each dimension and development stage of every migration pillar.
2. Definition of the target TO-BE profile of the manufacturing enterprise: The future vision and desired profile of the manufacturing enterprise are defined, considering links to business and competitive priorities. This target profile is then mapped onto each dimension and development stage of the 6P pillars.

**Figure 10:**
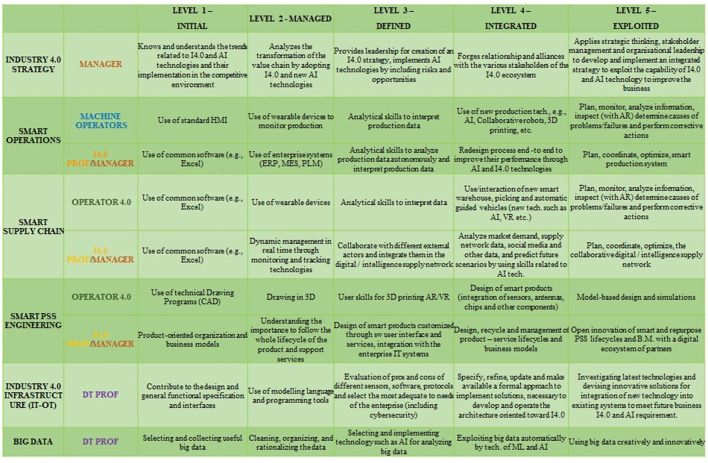
6Ps-the people dimension.

The six dimensions of analysis or pillars (referred to as “6Ps”) include product, process, platform, people, partnership, and performance, categorized into three technical and three socio-business pillars. As displayed in Figure 10, each pillar P is composed of at least six different dimensions of analysis of Industry 4.0 (rows). Each analysis dimension is broken down into five sequential development stages (columns) from the least to the most advanced one with respect to Industry 4.0 and AI adoption.

Since the original 6Ps methodology lacked specific dimensions for AI and Explainability, the XMANAI project introduced four new dimensions under the People pillar to comprehensively evaluate AI, especially XAI, implementation and its implications:

- *Teaming:* Focuses on the interaction between humans and AI when performing tasks together, ranging from no interaction to advanced interaction.
- *AI Integration:* Assesses the maturity of AI integration within production processes and its impact on the workforce.
- *Explainability:* Evaluates the transparency of AI models implemented and the extent to which human users can understand and trust AI decisions.
- *AI Development:* Analyzes how AI, including XAI, is developed and introduced within organizations, considering internal development, acquisitions from ICT providers, or collaborative research and development.

The People pillar has been identified as the most suitable for introducing dimensions related to explainability since it impacts the trustworthiness and understandability of algorithms, particularly affecting workers' interactions with AI. The XMANAI project uses these dimensions to measure the impact of explainability on the four manufacturing demonstrators.

To perform the evaluation, an online questionnaire is used, capturing both the AS-IS and TO-BE profiles before and after the adoption of the XMANAI platform. The questionnaire was followed by one-to-one meetings with the demonstrators to validate the answers and collect additional details.

**Figure 11:**
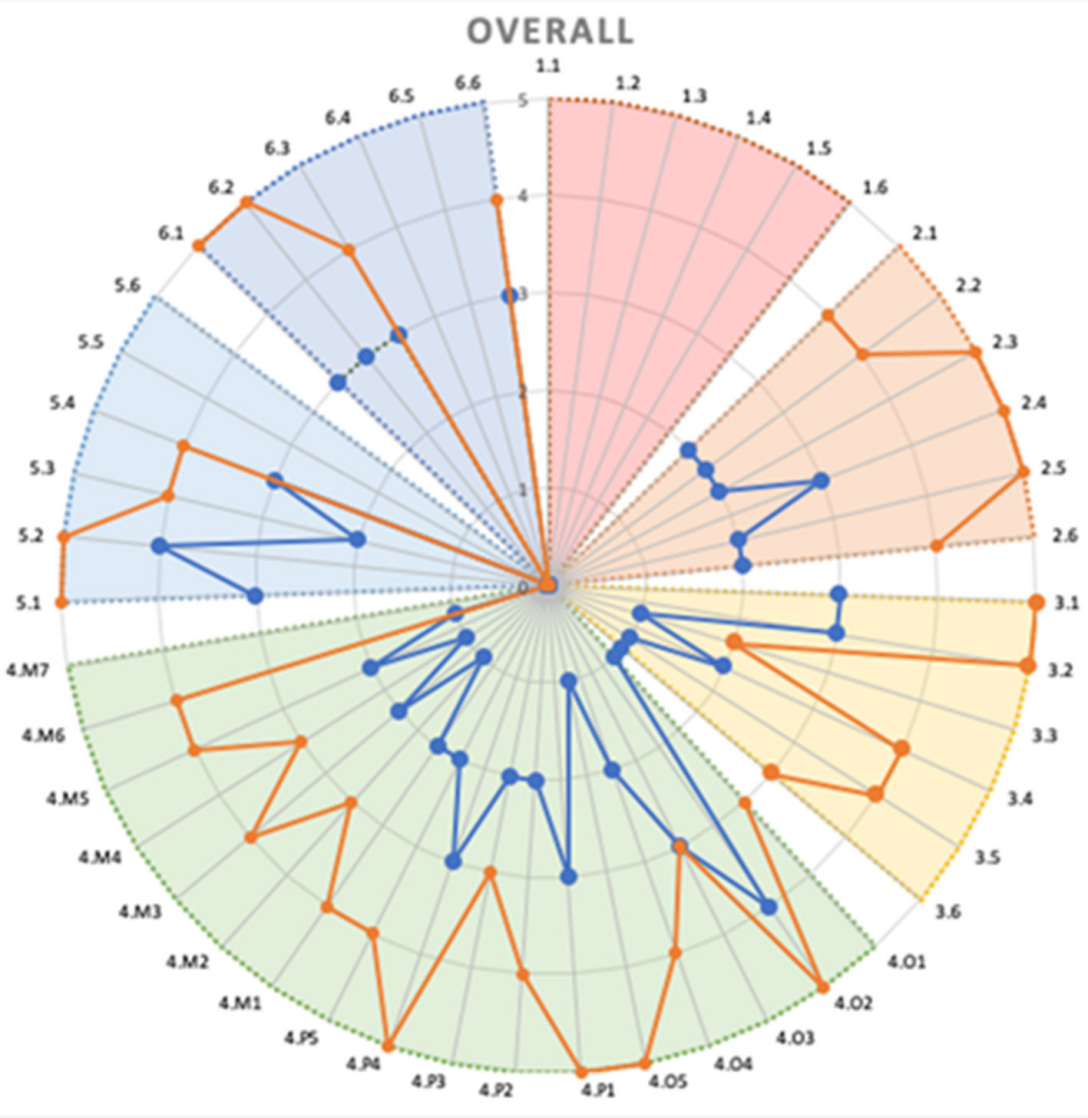
6Ps overall radar chart-an example.

**Figure 12:**
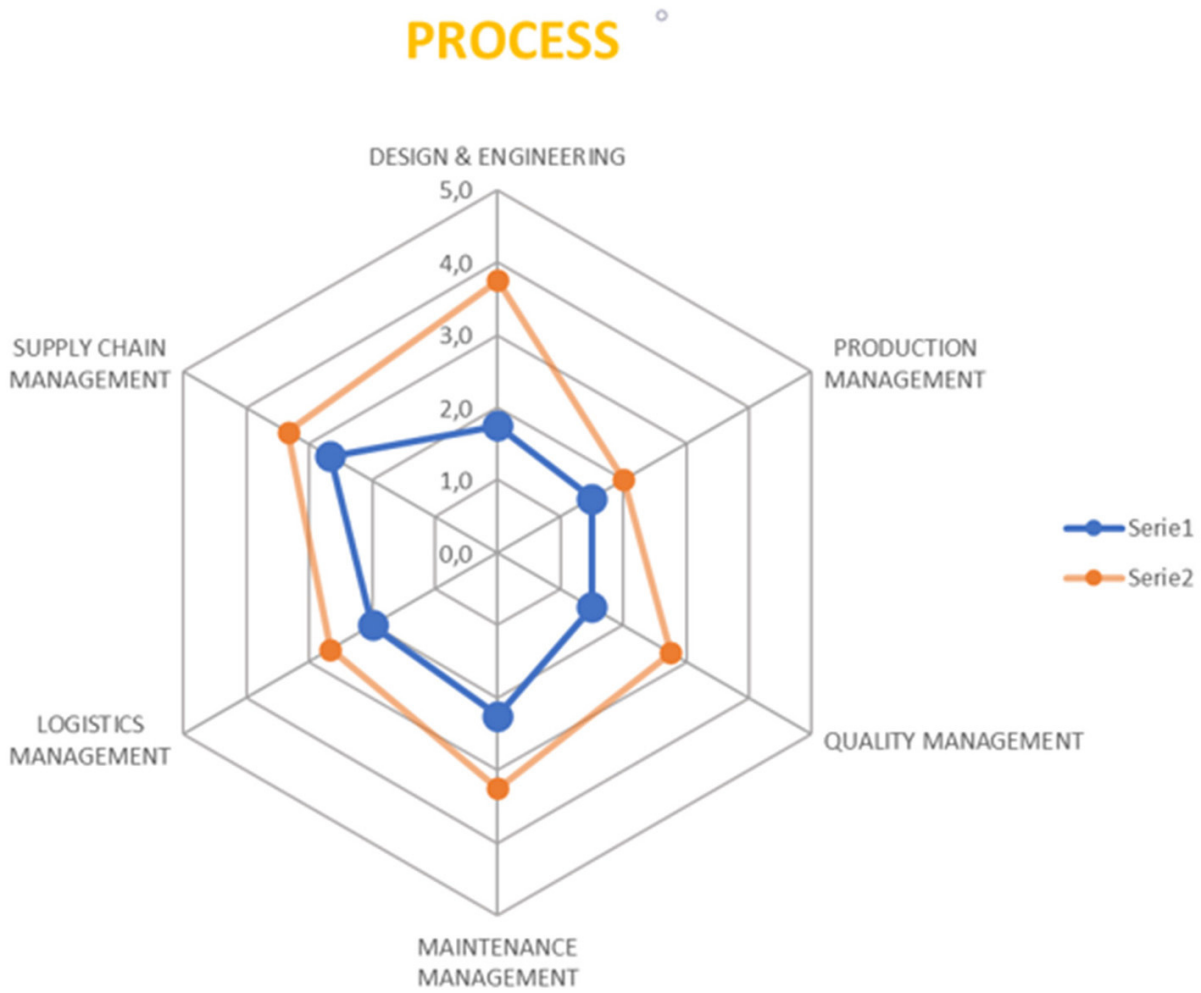
The Process pillar radar chart-an example.

Based on the analysis of the output from the questionnaire and the meetings, and taking into account the six pillars, a radar chart has been generated, comparing the AS-IS and TO-BE profiles. These charts practically visualize the impact on various aspects of the demonstrators, aiming to understand which areas are most affected by the adoption of the XMANAI platform. Two exemplary 6Ps radar charts are presented in Figures 11, 12, displaying the overall impact and the impact on Process pillar respectively.

This evaluation framework allows us to produce a comprehensive report showcasing the measurable impact of the XMANAI platform on the pilots. This is essential for understanding the effectiveness of the XAI features and how they enhance the manufacturing processes and interactions between humans and AI.

### A novel method for assessing business added value with Fuzzy Cognitive Maps

While the previous section introduced the extended 6P Methodology evaluation framework, which considers the socio-business dimensions, including the People pillar, to assess the impact of Explainable AI on the manufacturing demonstrators, this section, proposes a novel method for assessing the business added value of AI systems in Industry 5.0. While technical performance metrics are crucial, they do not provide a complete picture of the system's impact on business objectives. The inclusion of the People pillar, which can be seen as representing business perspectives, acknowledges the significance of considering human-AI interaction and the effect of explainability on workers. The proposed framework aims to bridge this gap by providing a systematic approach that considers both technical performance metrics and business objectives. The framework incorporates XAI and utilizes Fuzzy Cognitive Maps (FCMs) to predict Key Performance Indicators (KPIs) based on a comprehensive set of AI and XAI metrics. The methodology involves developing an FCM model that represents the causal-effect relationships between the various concepts and simulating different scenarios to evaluate the impact on KPI values. The proposed framework provides valuable insights for decision-making and resource allocation, ultimately leading to optimized business value.

#### Evaluation framework

The theoretical proposed validation framework integrates various technical and business perspectives to predict KPIs. For instance, it could be considered as AI metrics, such as accuracy (based on R2 score, Mean Absolute Percentage Error (MAPE) or other metrics), precision, recall, response time etc., as well as XAI metrics the AI Teaming (the interaction between humans and AI, and how humans and AI work together to perform tasks), the Explainability (the level of transparency of the AI models implemented so that human users will be able to understand and trust decisions), the AI development (the maturity of a process of integration of AI within production) etc. FCMs through scenario analysis employ expert knowledge to construct a model depicting causal-effect relationships among concepts (inputs and outputs). This methodology facilitates the evaluation of how various variables impact business outcomes by enabling the exploration of “what-if” scenarios and the assessment of changes in input variables (AI and XAI metrics) and their effects on outputs (KPIs). The choice of FCMs for the validation framework has multiple advantages (Kosko, 1991): (a) Modeling Complexity: FCMs are ideal for complex systems with numerous interconnected variables and cause-effect relationships, facilitating the representation of intricate technical and business metric interactions. (These models can be obtained through various methods (Özesmi and Özesmi, 2004), including (1) questionnaires, (2) extraction from written texts, (3) drawing from data depicting causal relationships, or (4) direct creation through interviews with experts who construct them), (b) Quantitative Translation: FCMs quantitatively translate qualitative expert insights into numerical data, enabling simulations and KPI predictions, (c) Scenario Analysis: FCMs excel in scenario analysis, allowing researchers to explore variable impacts on KPIs, aiding decision-making and optimization, (d) Efficiency and Reproducibility: FCMs provide an efficient, reproducible method for evaluating AI system impact without repeated interviews or data collection and (e) Visual Clarity: FCMs offer visual representations of causal relationships, enhancing understanding for researchers and stakeholders.

Moreover, this methodology involves four main steps (Dikopoulou, 2021): determining input and output features, categorizing the concepts, specifying causal-effect relationships and simulating the FCM to predict the output values. Specifically, the first step in the methodology is to identify the relevant input and output features that contribute to the evaluation of business value. The second step involves categorizing the concepts into groups. These categories provide a way to define the qualitative levels or ranges of each concept, such as Low, Medium, and High. Experts further refine the categories by aligning them with numerical values within a range of 0.1 to 1. This process allows for the quantification of the qualitative linguistic classes into numerical representations. Next, the causal-effect relationships between the concepts are specified in a range of−1 and 1. This step requires expert knowledge and understanding of the domain to determine how changes in one concept may influence others. Generally, three possible types of causal relationships between concepts can be met (Kosko, 1986):

- Positive causality between two concepts Ci and Cj in which an increase (decrease) on the value of Ci leads to an increase (decrease) on the value of Cj.
- Negative causality indicates in which an increase (decrease) on the value of Ci leads to a decrease (increase) on the value of Cj.
- No causal relationship between Ci and Cj.

Finally, the FCM is simulated to predict KPI values based on different scenarios. The simulation process uses an inference rule. The well-known inference rule is called rescale and it is denoted in (1). This rule updates the values of all concepts of the graph model using a calculation rule such as sigmoid (2). This iterative process continues until an equilibrium point is reached, providing insights into how different input features influence the business value.


(1) $$V_{i}^{\left(\kappa +1\right)}=\;\left((2*V_{i}^{\left(\kappa \right)}-1)+\sum_{j=1,\;j\neq i}^{n}w_{ji}*(2*V_{j}^{\left(\kappa \right)}-1)\right)$$

(2) $$\begin{array}{l} f\left(x\right)=\frac{1}{1+e^{-\lambda x}} \end{array}$$

By considering these metrics in conjunction with subjective KPIs, the proposed framework enables decision-makers to make informed choices and optimize the overall business value generated by their AI systems.

#### Scenario analysis

**Figure 13:**
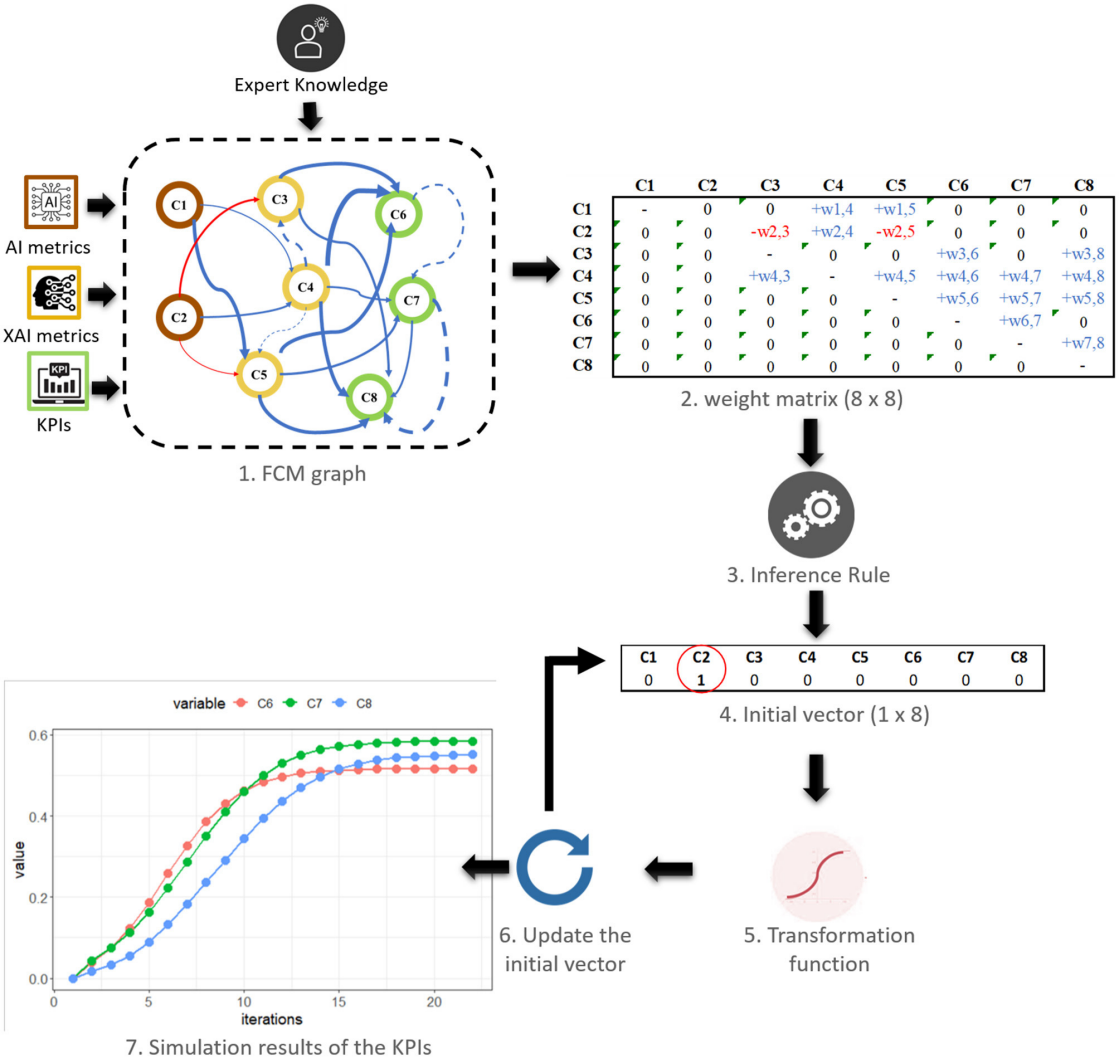
The simulation procedure of the FCM graph model activating in the initial vector one input concepts (C2-MAPE).

It is important to note that each FCM model is defined by a squared weight matrix that captures the causal-effect relationships between concepts. The weight matrix serves as a fundamental component of the FCM, encoding the strength and directionality of the connections among the concepts. Figure 13 illustrates AI metrics, depicted in brown color, encompassing the R2 score (C1) and the Mean Absolute Percentage Error (MAPE) (C2). On the other hand, the XAI metrics, represented in yellow color, consist of Teaming (C3), Explainability (C4), and AI development (C5). The output KPIs concepts, denoted in green color, reflect essential business metrics, including Product Quality (C6), Customer Satisfaction (C7), and Sales Growth Rate (C8). To simulate the scenario, the initial vector is set with specific values for selected input concepts. Blue and red arcs indicate positive and negative relations between concepts, respectively. Wider and more saturated arcs represent stronger causal-effect relationships. To predict the KPI values based on the activated scenario, we employed the “fcm” R package (Dikopoulou and Papageorgiou, 2017; Dikopoulou et al., 2018); utilizing the fcm.infer function to simulate the FCM graph model and generate the KPI outcomes.

For instance, consider a scenario in which the accuracy of the AI model is intentionally minimized by activating the MAPE (C2) metric to assess its impact on the KPIs. The FCM model, which represents the causal-effect relationships between the concepts, is then simulated. During the simulation, the values of the concepts are updated based on their causal-effect relationships. For example, an increase in MAPE may lead to a decrease in AI Teaming, as errors can cause frustration and distrust among team members. It may also lead to a greater need for explainability, as users may require more information to understand and prevent errors. These relationships influence not significant the KPIs, product quality, customer satisfaction, and sales growth rate, suggesting that other factors (the direct) may have a stronger effect on these business indicators.

The simulation results demonstrated that an increase in C2 metric had a detrimental effect on the values of the KPIs. This negative impact was observed because the concept C2 had a negative influence on both C3 and C5. Consequently, an increase in the C2 metric resulted in decreased values of the KPIs associated with C3 and C5. However, it is important to note that the KPIs values were not reduced to zero despite the negative effect of C2. This was primarily due to the positive influence of C2 on the concept C4, which in turn had a positive influence on the KPIs. The simulation process was carried out until the 22 step, and the resulting predicted values of the KPIs were as follows: C6 had the lowest value at 0.52 (red), followed by C8 at 0.55 (blue), while the KPI with the highest medium effect was observed on C7, reaching a value of 0.58 (green). However, it is important to acknowledge that the FCM map and the resulting outcomes do not accurately reflect a real-world problem. The purpose of the simulation was to illustrate the behavior of the model within a controlled scenario. Therefore, the presented values should not be interpreted as indicative of actual business performance.

By simulating different scenarios and observing the resulting impacts, decision-makers can gain insights into the complex causal relationships between the input concepts and output KPIs. They can evaluate the trade-offs between different factors and make informed decisions to optimize the overall performance of the AI system.

## Discussion and future work

### XMANAI platform – challenges and lessons learned

The major challenges faced during the design phase of XMANAI platform's reference architecture can be divided into two main axes: (a) the integration challenges and (b) the XAI challenges.

From the integration perspective, the complexity of the integration process is amplified due to the XMANAI platform's reference architecture comprising three tiers situated in different network locations (cloud vs. on-premise installations). These tiers incorporate eight distinct service bundles with a total of 12 different components. Successful and seamless integration across these layers is essential to enable proper intercommunication and interaction for the desired platform functionalities. However, the integration process is complicated by the utilization of diverse state-of-the-art technologies and tools within each service bundle. These technologies and tools have varying levels of maturity, specific programming language peculiarities, and limitations, further adding to the complexity of integration. Additionally, AI pipelines require modularity in their underlying components, but they also exhibit strong dependencies necessary for effective management of the machine learning development and deployment lifecycle, posing an additional challenge.

From the perspective of XAI, the collaborative preparation of AI for business problems becomes increasingly complex, as it involves iterative exchanges between data scientists and business users. Ensuring that XAI pipeline results are comprehensive and leveraging relevant manufacturing data requires constant iterations. Moreover, presenting AI insights in an understandable and explicit manner that caters to the perspective of the business user is crucial. However, the current state of available XAI solutions and approaches is not yet mature enough, necessitating further research in many areas. The formalization of the nature, structure, and format of explanations, as well as the definition of concrete metrics to assess their utility and added value for different stakeholders, remains an open challenge (Kim et al., 2021). Furthermore, the trade-off between model explainability and performance is yet to be effectively resolved. It is worth noting that more understandable models may sacrifice accuracy (Došilović et al., 2018). Deep learning models, in particular, present challenges in achieving explainability (Barredo Arrieta et al., 2020). Non-image, non-text, and other heterogeneous data types such as sequences, graphs, and spatio-temporal data also lack comprehensive explanations within AI (Saeed and Omlin, 2023). While considerable research efforts have focused on the development lifecycle of machine learning models, including training, deployment, and management, there is a lack of support for explainability features in this lifecycle. Additionally, further research is needed to address scalability issues in existing XAI models and methods, as the computational requirements for multivariable problems can be significant. Overall, the design phase of the XMANAI platform faced notable challenges in both integration and XAI, requiring careful consideration and additional research to efficiently overcome these obstacles and achieve a successful implementation.

### Practical implications and future perspectives of the proposed validation framework

The proposed validation framework addresses the gap in the literature by integrating technical and business perspectives for evaluating the business value of AI systems in Industry 5.0. By utilizing FCMs and incorporating AI and XAI metrics, the framework offers valuable insights for organizations seeking to optimize their operations. The integration of technical performance metrics, XAI metrics, and subjective KPIs allows decision-makers to consider a comprehensive set of factors when evaluating the impact of AI systems on business value. Generally, the framework provides a promising approach to effectively evaluate and optimize AI systems in Industry 5.0, leading to enhanced business value and competitiveness. In the subsequent sections of our research, we will examine additional, alternate practices and frameworks for assessing business-added value, taking into account their specific advantages and disadvantages. Our objective is to provide a well-rounded analysis that acknowledges the diversity of all available approaches. However, further research and operational validation to the XMANAI use-cases are necessary to fully assess the effectiveness and applicability of the proposed framework in additional diverse industry contexts (beyond the scope of the four industrial cases where it has been already applied). Nonetheless, this work provides a significant step toward bridging the gap in the literature regarding the evaluation of business value in Industry 5.0.

## Conclusions

This paper presented the XMANAI concrete approach as a solution to mitigate the challenges associated with transparency and interpretability in black box AI models within the manufacturing industry. By leveraging advancements in Explainable Artificial Intelligence (XAI) and exploiting the functionalities of the XMANAI platform, “glass box” AI models have been successfully constructed to exhibit a high degree of transparency while maintaining commendable performance levels. The XMANAI approach has overall contributed to the long-standing “transparency paradox” of AI by fostering transparent and trustworthy collaboration between human operators and machine systems.

In addition, the paper highlighted that the XMANAI platform has not only tackled technical challenges related to transparency but has also catered to the industry-specific requirements of manufacturing, encompassing vital aspects such as lifecycle management, security, and the secure exchange of AI assets. The platform demonstrated its significant potential in realizing transparency in manufacturing decision-making processes, fostering trust and collaboration between human operators and AI systems, enhancing operational efficiency, and optimizing overall business value. Through a synergistic incorporation of advanced Explainable AI techniques and the sophisticated features of the XMANAI platform, demonstrators within the manufacturing industry have been empowered to embrace and deploy AI technologies while ensuring transparency, interpretability, and a human-centric approach to decision-making.

Overall, this paper has emphasized the importance of transparency and explainability within the manufacturing industry and demonstrated how the XMANAI approach has comprehensively contributed to addressing these critical challenges. The outcomes clearly illustrated the significant potential of the adopted strategy in facilitating transparent and trustworthy collaboration, optimizing decision-making processes, and unlocking the full benefits of AI in the manufacturing domain, thus paving the way for a future where humans and AI systems work hand in hand toward mutual success.

## Data availability statement

The data analyzed in this study is subject to the following licenses/restrictions: The datasets used in the development and validation of the XAI models and in general the data handled in the XMANAI platform belong to the XMANAI demonstrators and are therefore proprietary, only to be handled by the XMANAI scientists within the scope of each demonstrator. Requests to access these datasets can be made by authenticated users of the XMANAI platform at: https://xaipd.ai4manufacturing.eu/.

## Author contributions

CA: Writing—original draft, Writing—review & editing, Investigation, Project administration. ZD: Writing—original draft, Writing—review & editing, Conceptualization, Methodology, Software, Validation. EL: Writing—original draft, Writing—review & editing. KP: Conceptualization, Software, Writing—original draft. SP: Conceptualization, Software, Writing—original draft. RB: Conceptualization, Data curation, Software, Visualization, Writing—original draft. SRe: Conceptualization, Data curation, Investigation, Software, Writing—original draft, Writing—review & editing. JH: Data curation, Investigation, Software, Writing— original draft. EB: Conceptualization, Data curation, Investigation, Supervision, Validation, Writing—original draft. FL: Conceptualization, Formal analysis, Investigation, Project administration, Supervision, Writing—original draft. SRo: Investigation, Methodology, Writing—original draft, Validation. VG: Software, Writing—original draft.

## Footnotes

^1^ISO 10303-1:2021(en) Industrial automation systems and integration — Product data representation and exchange; https://www.iso.org/obp/ui/#iso:std:iso:10303:-1:ed-2:v1:en.
